# Chronic Histiocytic Intervillositis: From Microscopic Placental Pathology to Clinical Impact—Case Series

**DOI:** 10.1155/crip/9628969

**Published:** 2026-05-09

**Authors:** Verónica Melgar-Cossich B, Anggi M. Vélez-Bohórquez, Olaya-C Mercedes

**Affiliations:** ^1^ School of Medicine, Pontificia Universidad Javeriana, and Hospital Universitario San Ignacio, Bogota, Colombia, javeriana.edu.co; ^2^ Pathology Department, Pontificia Universidad Javeriana, and Hospital Universitario San Ignacio, Bogota, Colombia, javeriana.edu.co

**Keywords:** chronic histiocytic intervillositis, COVID-19, macrophages, placenta, spontaneous abortion, stillbirth

## Abstract

Chronic histiocytic intervillositis (CHI) is a rare placental disorder characterized by an irregular and diffuse infiltration of the intervillous space by a monomorphic population of macrophages and monocytes. CHI is associated with adverse pregnancy outcomes, including intrauterine fetal demise, miscarriage, fetal growth restriction (FGR), and preterm birth. Its etiology remains unknown, and its pathophysiology is not fully understood; however, an underlying immune disorder targeting the semiallogeneic fetus has been proposed. Notably, CHI has undergone changes following the COVID‐19 pandemic, now recognized as part of the SARS‐CoV‐2 placentitis triad.

In this case series, we describe two pregnancies affected by CHI, highlighting their clinical relevance due to the immediate neonatal implications and the impact on maternal obstetric prognosis.

## 1. Introduction

Chronic histiocytic intervillositis (CHI) is a rare placental inflammatory disorder with an estimated incidence of 5 per 10,000 pregnancies [1, 2]. It can occur in any trimester and is associated with severe obstetric outcomes, including fetal growth restriction (FGR, 42%–61%), second‐trimester miscarriage (24%), and stillbirth [2–4]. Recurrence in subsequent pregnancies ranges from 25% to 100% [1]. Its pathogenesis remains incompletely understood, although alloimmune mechanisms have been proposed, based on maternal antibodies directed against paternal HLA antigens. Prothrombotic phenomena may contribute to perivillous fibrin deposition [1, 2, 5]. During the COVID‐19 pandemic, CHI incidence increased, and it is now recognized within the spectrum of SARS‐CoV‐2 placentitis. CHI is diagnosed exclusively postpartum via histopathology, though diagnostic criteria vary across studies [6, 7].

## 2. Case Presentation

*Case 1*: A 29‐year‐old gravida 2, para 1 woman with pregestational obesity and a history of cesarean section for breech presentation presented at 38.2 weeks of gestation with gestational hypertension and absent fetal movements. On admission, blood pressure was borderline (138/95 and 130/92 mmHg). Fetal heart rate was 148 bpm. A repeat cesarean section was performed, delivering a female neonate with Apgar scores of 8 and 9 at 1 and 10 min, respectively. The Ballard score corresponded to 39 weeks, and spontaneous neonatal adaptation was observed. The newborn weighed 2836 g (21.7th percentile, INTERGROWTH‐21st) and measured 36.5 cm. Minor congenital anomalies were identified: a left preauricular appendage and a dermoid cyst on the labia majora. During hospitalization, the neonate developed hypernatremic dehydration, excessive weight loss (13%), and jaundice secondary to ABO incompatibility without hemolysis (maternal blood type O+, paternal and neonatal A+). Indirect bilirubin levels were 11.75 mg/dL on Day 1 and 0.30 mg/dL on Day 2. The infant was discharged on Day 6 in stable condition and was clinically well at 9‐day follow‐up. Serological screening for *Toxoplasma gondii*, HIV, *Treponema pallidum*, and hepatitis B was negative. The mother′s postoperative course was uneventful. She underwent concurrent tubal sterilization and was discharged on Day 2 with stable blood pressure.

Placental examination revealed a specimen within the 10th–25th percentile for weight and a trivascular, hypercoiled, marginally inserted umbilical cord (coiling index 0.68; reference range 0.07–0.3). Microscopy demonstrated a persistent right umbilical vein and a villous parenchyma with marked intervillous hypercellularity due to histiocytic infiltration (≈40%), along with extensive perivillous fibrin deposition exceeding 30%. Special stains (Ziehl–Neelsen, PAS, and silver) and immunohistochemistry for cytomegalovirus (CMV) and HSV‐1/2 were negative, excluding infectious etiologies.

*Case 2*: A 21‐year‐old primigravida with a dichorionic diamniotic twin pregnancy at 34.2 weeks was referred for threatened preterm labor and preeclampsia without severe features. She had no relevant medical history. Serological screening for rubella, HIV, CMV, and *Toxoplasma gondii* was negative. On admission, she was in the latent phase of labor with 1 cm cervical dilation and had concomitant hypochromic microcytic anemia. Fetal monitoring revealed recurrent profound decelerations, prompting urgent cesarean delivery, during which severe oligohydramnios was noted.

Two preterm female neonates with FGR were delivered. The first twin had an Apgar score of 0 at 1 min, requiring advanced resuscitation with positive pressure ventilation, orotracheal intubation, and mechanical ventilation. Umbilical cord gases suggested perinatal asphyxia; by 10 min, her Apgar score improved to 8. She weighed 1500 g (2.3rd percentile, INTERGROWTH‐21st) and measured 40 cm. The second twin had Apgar scores of 2 and 9 at 1 and 10 min, respectively. She weighed 1518 g (1.7th percentile) and measured 45 cm. She required positive pressure ventilation followed by CPAP. Umbilical cord gases also indicated perinatal asphyxia.

During hospitalization, both neonates developed pneumonia and myocarditis/pericarditis attributed to COVID‐19, along with complications of prematurity including surfactant deficiency, metabolic acidosis, hyperlactatemia, transient immunodeficiency, feeding immaturity, hypoglycemia, jaundice, and intraventricular hemorrhage (in one twin). One infant developed mild pulmonary hypertension; the other had right femoral vein thrombosis. Serological testing for STORCH infections (syphilis, HIV, hepatitis B, rubella, and CMV) was negative in both.

SARS‐CoV‐2 infection was confirmed by PCR in the twins and subsequently in both parents. The mother′s postoperative recovery was initially uneventful; however, she required readmission after testing positive for SARS‐CoV‐2. The twins were discharged on Days 34 and 40 of life, respectively, in stable condition, breastfeeding successfully, and without respiratory compromise.

**Figure 1:**
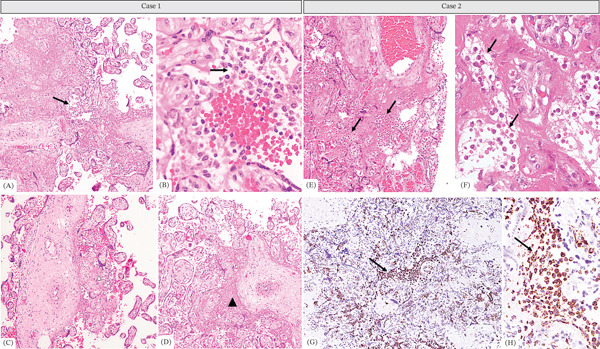
Case 1: (A, C, D) Chorionic villi of the third trimester with excess perivillous fibrin (arrowhead) and in the intervillous spaces, hypercellular appearance (arrow). H&E 4×. (B) Close‐up of the intervillous infiltrate which has a histiocytic appearance. H&E 40×. Case 2: (E) Chorionic villi of the third trimester with excess perivillous and intervillous fibrin deposition and increased intervillous cellularity (arrows). H&E 10×. (F) Close‐up of the intervillous infiltrate which has a histiocytic appearance. Necrosis of the syncytiotrophoblast is also observed. H&E 40×. (G, H) Immunohistochemistry with CD68 confirming the histiocytic origin of the intervillous inflammatory infiltrate (arrows) (4× and 40×, respectively).

Placental examination showed a dichorionic diamniotic placenta weighing below the third percentile. One umbilical cord had a normal central insertion, the other a velamentous insertion; both demonstrated normal three‐vessel architecture and left‐handed coiling. Microscopic analysis revealed extensive intervillous infiltration by histiocytes (≈80%), associated with > 30% perivillous fibrin deposition and trophoblastic necrosis. Immunohistochemistry confirmed CD68‐positive intervillous histiocytosis, while CMV and HSV‐1/2 were negative (Figure 1).

## 3. Discussion

CHI is characterized by intervillous infiltration of mononuclear macrophages, often accompanied by perivillous fibrin deposition, which impairs maternofetal exchange and predisposes to adverse perinatal outcomes [7, 8]. Its prevalence has been reported at 0.8% in early fetal demise and 0.6% in second‐ and third‐trimester placentas with pathological findings [3]. In our pathology service, CHI was identified in 0.42% of 710 examined placentas. Pregnancies beyond 22 weeks are particularly vulnerable, exhibiting high rates of FGR (62%) and fetal death (46%) [8–10]. Despite these risks, all three neonates in our series survived. Notably, the first case belonged to the relatively small subgroup of infants born alive with appropriate birth weight.

The pathophysiology of CHI remains incompletely understood [9]. Alloimmune mechanisms are supported by the predominance of CD68+ macrophages with M2 polarization and CR4 overexpression, along with mixed CD4+ and CD8+ lymphocytes [11]. These features distinguish CHI from villitis of unknown etiology [11]. Prothrombotic processes may also contribute to the extensive perivillous fibrin deposition [1, 2, 5]. Some authors classify CHI as a form of chronic intervillositis of unknown etiology, with intervillous histiocytes mixed with lymphocytes, often associated with villitis [11].

Historically, CHI has been linked to infectious agents, including malaria. More recently, SARS‐CoV‐2 has emerged as a potential trigger [5, 12]. SARS‐CoV‐2 placentitis is characterized by a triad of mononuclear intervillous infiltrates, syncytiotrophoblast necrosis, and massive perivillous fibrin deposition [13]. Placentas demonstrate strong syncytiotrophoblast immunoreactivity for SARS‐CoV‐2 [14], suggesting viral compromise of the vasculosyncytial membrane, a critical structure for maternal–fetal exchange. Notably, the severity of placental lesions does not always correlate with maternal disease severity [15].

In our cases, neonatal SARS‐CoV‐2 infection was diagnosed postnatally, with asymptomatic parents at delivery, leaving the timing of transmission uncertain. This highlights the possibility of subclinical maternal infection affecting the placenta and neonates. Neonatal complications included perinatal asphyxia, respiratory compromise, myocarditis/pericarditis, and complications of prematurity, emphasizing the significant morbidity associated with CHI in the setting of viral exposure.

Recognition of CHI is clinically important due to its high recurrence risk and severe perinatal outcomes, with reported mortality rates approaching 80% [8]. A prior diagnosis identifies women as high risk in subsequent pregnancies [16]. In addition, the association with SARS‐CoV‐2 underscores the need for meticulous placental examination in infected or exposed pregnancies. The potential impact of viral infections on the maternal–fetal interface may inform perinatal monitoring strategies and guide counseling for future pregnancies. Although SARS‐CoV‐2‐associated intervillositis is histologically indistinguishable from classic CHI, it is important to note that SARS‐CoV‐2‐associated cases have not been shown to recur. Given the typically poor prognosis associated with CHI, it is essential that parents, obstetricians, and other treating professionals are aware that when this placental diagnosis is linked to viral infection, the prognosis differs, as recurrence is not expected.

Furthermore, the histopathological patterns observed in SARS‐CoV‐2 placentitis, including extensive intervillous histiocytosis, trophoblastic necrosis, and massive perivillous fibrin deposition, reinforce the hypothesis that viral infection may exacerbate pre‐existing immunological or thrombotic placental vulnerabilities. These findings provide insight into the interplay between infection, immune response, and placental function, with implications for both obstetric management and neonatal care. Future studies are warranted to clarify the mechanisms by which SARS‐CoV‐2 contributes to CHI and to identify potential biomarkers for early detection and risk stratification.

## Funding

This work was supported by the Pontificia Universidad Javeriana (10.13039/501100009543) and the Hospital Universitario San Ignacio.

## Ethics Statement

The ethics committee approved this study (FM‐CIE‐0264‐22). Cases were obtained from routine histologic examinations of placentas for which evaluation had been previously indicated; no patient identifiers were included, and no additional or special procedures were performed for inclusion in the study.

## Conflicts of Interest

The authors declare no conflicts of interest.

## Data Availability

The data that support the findings of this study are available on request from the corresponding author. The data are not publicly available due to privacy or ethical restrictions.
